# Decreased Anti-Inflammatory Responses to Vitamin D in Neonatal Neutrophils

**DOI:** 10.1155/2011/598345

**Published:** 2011-12-15

**Authors:** Daniel Hirsch, Faith E. Archer, Meera Joshi-Kale, Anna M. Vetrano, Barry Weinberger

**Affiliations:** Division of Neonatology, Department of Pediatrics, Robert Wood Johnson Medical School, University of Medicine and Dentistry of New Jersey, New Brunswick, NJ 08901, USA

## Abstract

Neutrophil activity is prolonged in newborns, suggesting decreased exposure and/or responses to immunosuppressive modulators, such as 1,25-hydroxyvitamin D_3_ (1,25-vit D_3_). We hypothesized that 1,25-vit D_3_ suppresses neutrophil activation and that this response is impaired in newborns. Consistent with this, 1,25-vit D_3_ decreased LPS-induced expression of macrophage inflammatory protein-1**β** and VEGF in adult, but not neonatal, neutrophils. Expression of vitamin D receptor (VDR) and 25-hydroxyvitamin D_3_-1**α**-hydroxylase was reduced in neonatal, relative to adult neutrophils. Moreover, 1,25-vit D_3_ induced VDR gene expression in activated adult, but not neonatal, neutrophils. 1,25-vit D_3_ also suppressed expression of cyclooxygenase-2 and induced expression of 5-lipoxygenase in LPS-exposed adult neutrophils, while neonatal cells were not affected. 1,25-vit D_3_ had no effect on respiratory burst in either adult or neonatal cells. Anti-inflammatory activity of vitamin D is impaired in neonatal neutrophils, and this may be due to decreased expression of VDR and 1**α**-hydroxylase. Insensitivity to 1,25-vit D_3_ may contribute to chronic inflammation in neonates.

## 1. Introduction

There is a high incidence of vitamin D insufficiency in neonates, particularly those who are exclusively breast fed [1]. After undergoing 25-hydroxylation in the liver, 25-hydroxyvitamin D_3_ is converted, via 25-hydroxyvitamin D_3_-1*α*-hydroxylase (1*α*-hydroxylase), to the active form 1,25-vit D_3_ in many cell types. The biologic effects of 1,25-vit D_3_ are primarily mediated via the nuclear transcription factor, vitamin D receptor (VDR), which triggers expression of vitamin D-responsive genes. While the importance of vitamin D in calcium and phosphate homeostasis is well known, recent studies have demonstrated that vitamin D has “nonclassical” effects, including an important role in downregulating immune responses. VDR and 1*α*-hydroxylase are expressed in monocytes, dendritic cells, T-lymphocytes, and granulocytes, suggesting that 1,25-vit D_3_ plays a role in differentiation, biosynthetic activity, and function of leukocytes [2, 3].

Circulating neutrophils are the main effector cells in innate immunity but can contribute to pathology if their activity is prolonged [4, 5]. Resolution of the neutrophilic inflammatory response is an active process involving downregulation of proinflammatory cytokines, upregulation of anti-inflammatory eicosanoids, decreased generation of reactive oxygen intermediates, and removal of the cells [6]. In this regard, we have previously shown that the clearance of neutrophils by apoptosis is impaired in neonates when compared to adults [7]. Prolonged viability of neonatal neutrophils is thought to play a role in chronic inflammatory disease because these cells release oxygen radicals, hydrolases, and inflammatory cytokines. This results in cytotoxicity, potentially contributing to the high incidence of neutrophil-mediated inflammatory diseases in newborns. 

Previous studies have suggested that vitamin D downregulates neutrophil function [8]. In these studies, we investigated the hypothesis that 1,25-vit D_3_ decreases production of inflammatory mediators and reactive oxygen intermediates in neutrophils and that this activity is impaired in neonatal neutrophils. We further hypothesized that reduced responses in neonates may be related to decreased expression of VDR and 1*α*-hydroxylase in neonatal neutrophils. Impaired responsiveness to 1,25-vit D_3_ may contribute to prolonged viability and activity of these cells, leading to increased susceptibility to chronic inflammatory diseases.

## 2. Materials and Methods

### 2.1. Reagents

RNA purification kits were purchased from Qiagen (Chatsworth, CA). Primers for RT-PCR were obtained from Integrated DNA Technologies (Coralville, IA). Reagents for RT-PCR were from Applied Biosystems (Foster City, CA). Amplex Red and horseradish peroxidase were from Molecular Probes (Carlsbad, CA), DMEM and dextran from Sigma Chemical Co. (St. Louis, MO), and Ficoll-paque from GE Healthcare (Piscataway, NJ). 7-actinomycin D (7-AAD) and cytometric bead array flex sets were from BD Biosciences (San Jose, CA). 1,25-vit D_3_ was obtained from Enzo Life Sciences (Plymouth Meeting, PA) and solubilized in ethanol at a stock concentration of 10^−4 ^M. The final concentration of 10^−7 ^M in cell culture was based on estimates of tissue concentrations and on previous studies demonstrating biologic effects at this concentration [8].

### 2.2. Subjects and Neutrophil Isolation

Studies were approved by the Institutional Review Board of Robert Wood Johnson Medical School and informed consent obtained from subjects. Umbilical cord blood was obtained from the placentas of term infants (≥37 wk gestation, *n* = 27) delivered by cesarean section without labor. Peripheral venous blood collected from healthy adults (*n* = 25) was used for comparison. Neonatal and adult samples were anticoagulated using heparin and processed immediately in parallel. Subjects were excluded for clinical evidence of infection, history of immunodeficiency, diabetes, pregnancy-induced hypertension, or exposure to tobacco or to medications that affect immune function. While subjects were also to be excluded if there was recent history of vitamin D intoxication (due to unusual dietary supplementation) or deficiency (diagnosed secondary to neonatal hypocalcemia), no prospective subjects met these criteria. Equivalent numbers of adult and neonatal samples were collected concurrently and uniformly throughout a single year, to minimize any potential seasonal effects. Neutrophils were isolated by dextran sedimentation and Ficoll gradient centrifugation, with >95% viability and purity confirmed by trypan staining. In preliminary experiments, 1,25-vit D_3_ did not affect neutrophil viability or apoptosis, as determined by flow cytometric measurement of propidium iodide or annexin V binding, respectively.

### 2.3. Analysis of mRNA Expression

Neutrophils were incubated with control medium (DMEM + 10% fetal bovine serum), bacterial lipopolysaccharide (LPS, 1 *μ*g/mL), and/or 1,25-vit D_3_ (10^−7 ^M) for 4 hr. LPS was used as an inflammatory stimulus because of the diverse CD14/TLR4-mediated responses that have been demonstrated in neutrophils. RNA was isolated using a RNeasy Minikit (Qiagen) and cDNA synthesized using a reverse transcription kit (Applied Biosystems, Foster City, CA). Real time PCR was performed using SYBR Green PCR Master Mix and amplified on an ABI Prism 7900 sequence detection system, using GAPDH as a standard. Full-length coding sequences for genes to be analyzed were obtained from GenBank. Primers were designed using Primer Express software and synthesized by Integrated DNA Technologies (Coralville, IA). The following primers were used: VDR: 5′-TCC CTG TCA CCA AGC TCA CA-3′; 5′-CAA CCA ACC CCT TAG ACC CAG-3′; 1*α*-hydroxylase: 5′-AGA CAT CCC AGG CCC CTC TA-3′; 5′-TTG CAG AAA AGT TCG GCC A-3′. 5-lipoxygenase (5-LOX): 5′-AGG GAG AAG CTG TCC GAG T-3′; 5′-GCA GAG GCC GTG AAG ATC AC-3′; cyclooxygenase-2 (COX-2): 5′-GCC TGA TGA TTG CCC GAC T-3′; 5′-GCT GGC CCT CGC TTA TGA TCT-3′.

### 2.4. Inflammatory Proteins

Neutrophils were incubated with control medium (DMEM + 10% fetal bovine serum), LPS (1 *μ*g/mL), and/or 1,25-vit D_3_ (10^−7 ^M) for 24 hr. Culture supernatants were incubated with premixed flex set beads coated with antibodies to macrophage inflammatory protein-1*β* (MIP-1*β*), IL-8, vascular endothelial growth factor (VEGF), and IL-1*β* in 96-well filtration plates. Premixed phycoerythrin-labeled detection reagent was then added to the wells and incubated in the dark for 2 hr. The bead-protein complexes were analyzed for fluorescence intensity using a BD FACSArray Bioanalyzer. Data were analyzed using BD FCAP software (v 2.0).

### 2.5. Measurement of Hydrogen Peroxide (H_**2**_O_**2**_) Production

Neutrophils were plated in 96-well dishes (5 × 10^4^cells/well). A reaction mixture (50 *μ*L) containing Amplex Red (25 *μ*M) and horseradish peroxidase (1.07 U/mL) was added to each well, followed by the inflammatory stimulus, phorbol 12-myristate 13-acetate (PMA, 500 nM), 1,25-vit D_3_ (10^−7 ^M), 1,25-vit-D_3_ + PMA, or medium control. Fluorescent product formation, indicative of H_2_O_2_, was measured spectrophotometrically at 1 min intervals for 30 min at 540 nm excitation and 590 nm emission on a Perkin Elmer HTS 7000 Bio Assay Reader [9].

### 2.6. Data Analysis

Comparison of cytokine levels and mRNA expression in neonates and adults was performed using the Wald-Wolfowitz Runs Test. In order to compare the values for treatments in same subjects, analyses for dependent measures were calculated using the Wilcoxon Matched Pairs Test. To compare multiple dependent samples derived from repeated measures of H_2_O_2_ levels over time (0, 10, 20, and 30 min), we performed Friedman ANOVA. These analyses were performed for each treatment condition in newborns and adults. Within-subject comparisons of treatment with PMA and PMA + 1,25-vit D3 at specified time points were performed using Wilcoxon Matched Pairs Tests. H_2_O_2_ generation by neonatal and adult neutrophils was compared using the Mann-Whitney U test.

## 3. Results and Discussion

**Figure 1:**
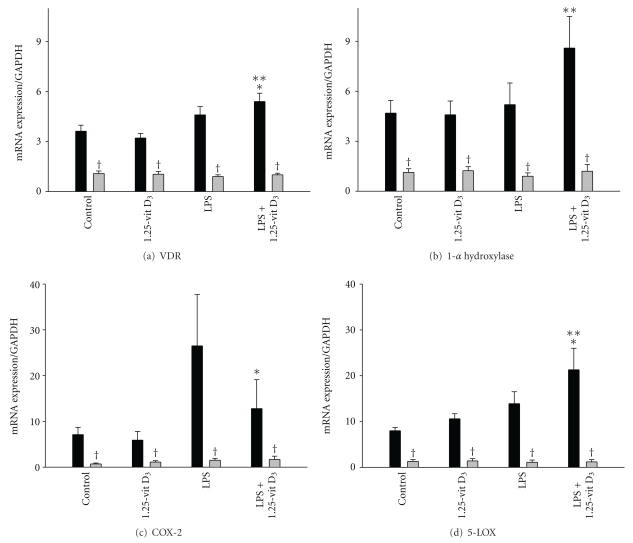
Effects of LPS and 1, 25-vit D_3_ on expression of VDR, 1-*α* hydroxylase, COX-2, and 5-LOX in neutrophils. Adult (black column) and neonatal (grey column) neutrophils were incubated with LPS (1 *μ*g/mL) and/or 1,25-Vit D_3 _(10^−7 ^M), or medium control for 4 h. mRNA expression of the (a) VDR, (b) 1-*α* hydroxylase, (c) COX-2, and (d) 5-LOX. Results were normalized to GAPDH expression. Each bar represents the mean ± SE (*n* = 7–11). *Significantly different (*P* < 0.05) from LPS; **significantly different (*P* < 0.05) from control; ^†^significantly different (*P* < 0.05) from adult.

In order to model bacterial-induced tissue inflammation, we analyzed the responses of LPS-treated adult and neonatal neutrophils to 1,25-vit D_3_. In initial studies, we compared the effects of 1,25-vit-D_3_, LPS, and LPS + 1,25-vit D_3_ on mRNA expression of genes involved in vitamin D metabolism and inflammation. Constitutive mRNA expression of VDR, 1-*α*-hydroxylase, 5-LOX, and COX-2 was decreased in neonatal, when compared to adult neutrophils (Figure 1). While 1,25-vit D_3_ or LPS alone did not significantly affect expression of these genes in either adult or neonatal cells, treatment with LPS + 1,25-vit D_3_ was associated with a significant induction of VDR in adult cells when compared to control or LPS alone. 1-*α*-hydroxylase expression was also increased in adult cells after exposure to LPS + 1,25-vit D_3_. In contrast, LPS + 1,25-vit D_3_ did not affect expression of VDR or 1-*α*-hydroxylase in neonatal neutrophils. In addition, 1,25-vit D_3_ induced 5-LOX and inhibited COX-2 gene expression in LPS-treated adult cells, while neonatal cells were not affected.

**Figure 2:**
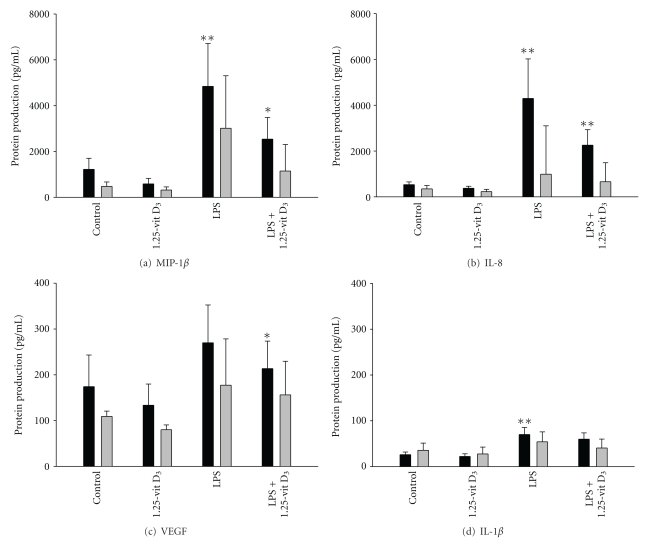
Effects of MEHP on inflammatory mediator production. Adult (black column) and neonatal (grey column) neutrophils were incubated in the presence or absence of LPS (1 *μ*g/mL) and/or 1,25-Vit D_3 _(10^−7 ^M) for 24 h. The inflammatory mediators (a) MIP-1*β*, (b) IL-8, (c) VEGF, and (d) IL-1*β* were measured in culture supernatants using cytometric bead array analysis. Each bar represents the mean ± SE (*n* = 4–11). *Significantly different (*P* < 0.05) from LPS; **significantly different (*P* < 0.05) from control.

In further studies, we compared the effects of 1,25-vit D_3_, LPS, and LPS + 1,25-vit D_3_ on the production of inflammatory cytokines and proteins by adult and neonatal neutrophils. Constitutive production of MIP-1*β*, IL-8, VEGF, and IL-1*β* was similar in adult and neonatal neutrophils (Figure 2). LPS induced production of MIP-1*β*, IL-8, and IL-1*β* adult cells, while neonatal cells were not affected. In adult neutrophils, LPS + 1,25-vit D_3_ significantly inhibited the generation of MIP-1*β* and VEGF when compared to LPS alone, and there was a trend toward decreased production of IL-8. In contrast, 1,25-vit D_3_ did not significantly affect production of these inflammatory proteins in LPS-treated neonatal neutrophils.

**Figure 3:**
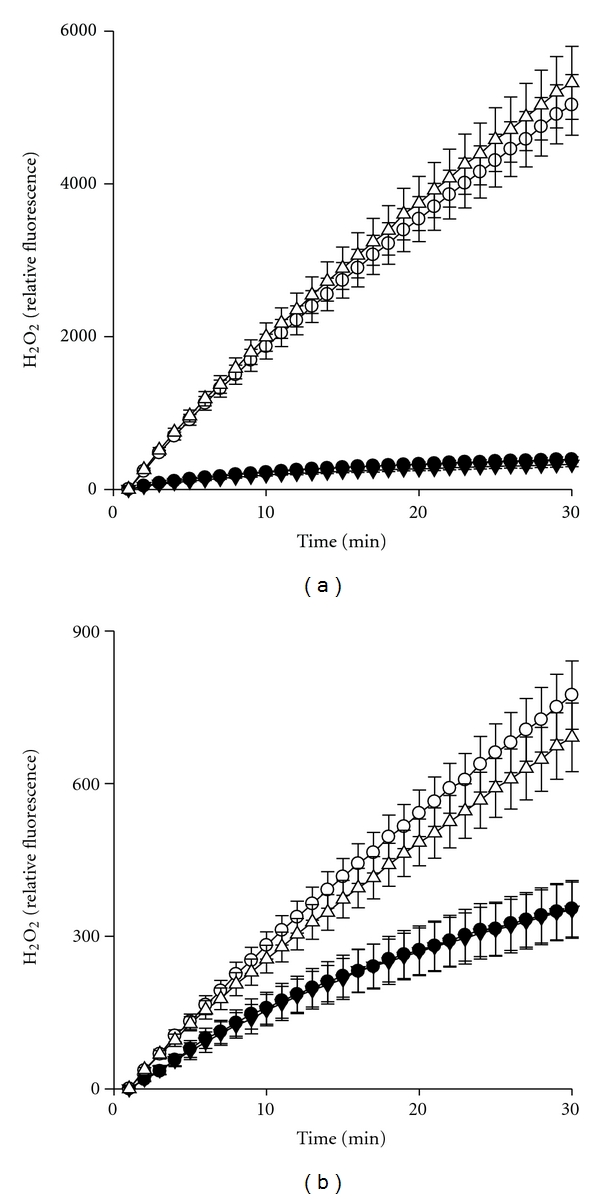
1,25 vit D_3_ does not suppress PMA-induced H_2_O_2_ generation in adult or neonatal neutrophils. Adult (a) and neonatal (b) neutrophils (5 × 10^4^ cells) were incubated with Amplex Red (25 *μ*M) and horseradish peroxidise and then treated with PMA (500 nM) or medium control, in the presence or absence of 1,25-vit D_3 _(10^−7^ M). H_2_O_2_ production was quantified at 1 min intervals for 30 min. Each point represents the mean ± SE (*n* = 6). Control, ∙; PMA, ◯  ; 1,25-Vit D_3_, ▾; PMA + 1,25-Vit D_3_, ▵.

We next compared the effects of 1,25-vit D_3_ on production of H_2_O_2_ in adult and neonatal neutrophils. Activated neutrophils secrete reactive oxygen intermediates in response to inflammatory mediators at sites of infection or tissue injury. In the absence of stimulation, both adult and neonatal cells generated detectible basal quantities of H_2_O_2_ (Figure 3). Consistent with previous studies, production of H_2_O_2_ in activated neutrophils was decreased in neutrophils from neonates, when compared to adults [10]. 1,25-vit D_3_ alone did not affect H_2_O_2_ production in either adult or neonatal cells. The inflammatory stimulant, PMA, induced respiratory burst activity in both adult and neonatal cells, but this response was less robust in neonatal cells. 1,25-vit D_3_ did not significantly affect PMA-stimulated H_2_O_2_ generation in either adult or neonatal neutrophils.

In these studies, we demonstrated that 1,25-vit D_3_ decreases specific inflammatory responses in mature circulating neutrophils and that this is impaired in neonatal cells. Previous findings on the effects of vitamin D on neutrophils have been inconsistent. For example, vitamin D induces expression of human cathelicidin antimicrobial peptide 18 [11], and vitamin D deficiency decreases TLR-induced cathelicidin expression in monocytes [12]. Therefore, vitamin D appears to play an important role in the integrity of the innate immune response. However, vitamin D also decreases production of IL-1*β* in neutrophils [8], and increasing evidence suggests that vitamin D can trigger resolution of inflammatory responses. 1,25-vit D_3_ inhibits differentiation, proliferation, and synthetic activity in T lymphocytes and induces a tolerogenic state in dendritic cells, suggesting that it suppresses adaptive immunity [13, 14]. While 1,25-vit D_3_ may promote differentiation and synthetic activity in monocytes and macrophages [15, 16], it also decreases proliferation and interleukin-2 production and inhibits production of IL-12 by downregulating NF-*κ*B activation in these cells [17, 18]. It also downregulates Toll-like receptor-(TLR-)2 and TLR-4 expression in human monocytes [19]. Our findings that 1,25-vit D_3_ decreases neutrophil activity support the idea that vitamin D plays a physiologic role in the suppression of immune responses.

Neonates are at particularly high risk for developing neutrophil-mediated inflammatory diseases, suggesting that there are developmental defects in signaling pathways that down regulate neutrophil activity and induce their clearance by macrophages. Consistent with this idea, our laboratory has previously shown that apoptosis is impaired in neutrophils from neonates when compared to adults [7]. In these studies, we have shown that responsiveness to vitamin D is also impaired in neonatal neutrophils. This may contribute to prolonged viability and activity of these cells, possibly contributing to chronic inflammatory disease secondary to the release of reactive oxygen species and inflammatory cytokines. Decreased responsiveness may be due to reduced expression of VDR. We found that adult neutrophils express VDR and that 1,25-vit D_3_ induces VDR expression in the presence of LPS. Induction of VDR by 1,25-vit D_3_ and LPS has also been observed previously in a human monocytic cell line [20]. In contrast, neonates expressed significantly lower levels of VDR, and this was not affected by vitamin D and LPS. We also found that expression of 1*α*-hydroxylase is significantly decreased in neonatal neutrophils, when compared to adults. 1*α*-hydroxylase is required for the generation of 1,25-vit D_3_, the biologically active form that triggers binding to response elements in the promoter regions of vitamin D responsive genes. Decreased expression of this enzyme suggests that activation of vitamin D may be impaired in neonatal leukocytes. In addition, 1,25-vit D_3_ induced 5-LOX and suppressed COX-2 gene expression in LPS-treated adult neutrophils, while neonatal cells were not affected. 5-LOX catalyzes the generation of anti-inflammatory eicosanoids such as lipoxins, while COX-2 exerts inflammatory effects via synthesis of prostaglandins [21]. The shift from generation of pro- to anti-inflammatory eicosanoids is thought to play a key role in signaling the resolution of neutrophil activity [22]. Impaired alteration of the ratio of 5-LOX/COX-2 gene expression in response to vitamin D may contribute to prolonged inflammation in neonates.

Previous in vitro and in vivo studies in humans have suggested that vitamin D is associated with decreased inflammatory cytokine production [8, 23]. To our knowledge, this is the first study to measure cytokine responses to vitamin D in neonatal neutrophils. We found that while vitamin D suppressed LPS-induced production of MIP-1*β* and VEGF in adult cells, it had no effect on the production of these mediators in neonatal cells. This is consistent with our finding that expression of VDR is impaired in neonatal neutrophils. Taken together, these data suggest that neonatal neutrophils have decreased capacity to synthesize 1,25-vit D_3_ and decreased responsiveness to its immunosuppressive effects.

Surprisingly, vitamin D did not reduce basal or PMA-induced generation of hydrogen peroxide in either adult or neonatal neutrophils. Consistent with this, previous investigators have reported that vitamin D restores chemotaxis and phagocytosis in vitamin D-deficient mice [24] and can facilitate bacterial killing in human cells [25]. Others have also shown that vitamin D induces expression of membrane and cytosolic components of NADPH-oxidase in monocyte-like cell lines [26]. The effects of vitamin D on respiratory burst activity are likely dependent on specifics of dose and endpoint, and this will be investigated in further studies.

## 4. Conclusions

We found that 1,25-vit D_3_ decreased LPS-induced expression of macrophage inflammatory protein-1*β* and VEGF in adult, but not neonatal, neutrophils. Our findings suggest that neonates may be resistant to the modulatory effects of vitamin D in neutrophilic inflammation and that this may be due to decreased expression of VDR and 1*α*-hydroxylase. Decreased responsiveness to vitamin D in neonates may compound the effects of vitamin D deficiency, which is now recognized as an important public health problem. Investigations of vitamin D status have revealed a high incidence of deficiency in both mothers and infants [26], and the American Academy of Pediatrics recently issued recommendations for increased dietary supplementation with vitamin D in neonates [27]. Understanding the immune-modulatory actions of vitamin D in neonates at the cellular level may strengthen efforts to adequately address this public health challenge, using strategies such as therapeutic levels of supplementation or treatment with synthetic VDR agonists.

## Abbreviation

1*α*-hydroxylase: 25-hydroxyvitamin D_3_-1*α*-hydroxylase
COX-2: Cyclooxygenase-2
5-LOX: 5-lipoxygenase
LPS: Bacterial lipopolysaccharide
MIP-1*β*: Macrophage inflammatory protein-1*β*
PMA: Phorbol 12-myristate 13-acetate
VEGF: Vascular endothelial growth factor
VDR: Vitamin D receptor
1,25-vit D_3_: 1,25-dihydroxycholecalciferol.
